# Pathways of Metastases from Primary Organs to the Ovaries

**DOI:** 10.1155/2011/612817

**Published:** 2011-09-11

**Authors:** Yukio Yamanishi, Masafumi Koshiyama, Megumi Ohnaka, Masashi Ueda, Shingo Ukita, Kenji Hishikawa, Michikazu Nagura, Tomoko Kim, Masaya Hirose, Hiroshi Ozasa, Tomoyuki Shirase

**Affiliations:** ^1^Department of Obstetrics and Gynecology, Otsu Red-Cross Hospital, 1-1-35 Nagara, Otsu, Shiga 520-0046, Japan; ^2^Department of Pathology, Otsu Red-Cross Hospital, 1-1-35 Nagara, Otsu, Shiga 520-0046, Japan

## Abstract

To investigate the metastatic pathways from the primary organs to the ovaries, we examined the microscopic findings from 18 original and 18 metastatic ovarian tumors carefully. In addition, we examined the immunohistochemical findings (Victoria blue stain for vascular invasion and D2-40 expression for lymphangio invasion) of metastatic ovarian tumors carefully. There were 4 (57%) ovarian lymphangio invasion cases in the 7 gastric cancers, but there were no cases in the 6 colorectal cancers (*P* < 0.05). There were 4 (67%) ovarian vascular invasion cases and one (17%) liver metastasis case in the 6 colorectal cancers, while there were no ovarian vascular invasions (*P* < 0.05) or no liver metastases in the 7 gastric cancers. The patients with metastatic ovarian tumors originating from distant organs who were treated at the same time as the original cancers had a significantly poorer prognosis than the patients with ovarian tumors treated later (*P* < 0.05). The rate of lymphatic metastasis from the stomach to the ovary was significantly higher than from the colon to the ovary. In addition we hypothesized that the rate of intravascular metastasis from the colorectum to the ovary was relatively higher than from the stomach to the ovary.

## 1. Introduction

Tumors metastasize to the ovaries from many organs, including the stomach, small intestine, colon, rectum, gall bladder, appendix, pancreas, breast, uterus, fallopian tube, and peritoneum. Tumors from the stomach, colon, and breast are the 3 most common neoplasms that metastasize to the ovary. Novak and Gray advanced the following criteria for Krukenberg tumors: (1) cancer in the ovary, (2) the presence of mucin-producing neoplastic signet-ring cells, and (3) ovarian stromal sarcomatoid proliferation  [1]. Krukenberg tumors are defined as gastrointestinal cancers that metastasize to the ovary. Recently, the term Krukenberg tumor has been used more widely and loosely to describe any metastatic lesion to the ovary. Metastatic tumors, except for Krukenberg tumors, show the various pathologic findings in the ovary  [2–4]. Recently, specific immunohistochemical methods have been tried in order to identify the primary neoplasm site [5, 6].

Many tumors arising from primary organs spread to the ovaries by various routes. Direct spread is one of the pathways for cancer invasion into adjacent organs. Spread from more distant sites is mainly via other routes, for example, blood vessels, lymphatics, and surface implantation from intra-abdominal cancers. There are many different pathways from distant origins, and sometimes these pathways are mixed. Several studies on the metastatic routes from the stomach have been reported [7, 8]. However, there have been few reports on the metastatic pathways from other organs to the ovary. 

 In this study, we investigated the metastatic pathways from primary organs to the ovaries by examining the microscopic findings from original and metastatic ovarian tumors carefully, by research into the intra-abdominal findings during surgery and by checking for positive or negative metastasis to the liver or lung clinically. In addition, we used immunohistochemical methods to detect vascular or lymphangio invasion to the ovary. Furthermore, we also compared the prognosis of metastatic ovarian tumors treated simultaneously as the primary cancers, versus the prognosis of metastatic ovarian tumors treated after the primary tumor.

## 2. Methods

Eighteen patients with pathologically confirmed metastatic ovarian tumor, who were treated between 2000 and 2009 at the Otsu Red Cross Hospital, were reviewed. During that period, we experienced 200 ovarian malignancies and 18 (9.0%) metastatic ovarian carcinomas. The origins of the 18 metastatic ovarian carcinomas were 7 gastric, 6 colorectal (2 ascending, 1 transverse, 1 sigmoid, and 2 rectal), 2 appendix, 1 small intestine, 1 gall duct, and 1 uterine corpus. We pathologically investigated any lymph node metastasis of the originating organs, lymphangio and vascular invasion of the ovary and direct invasion into the ovaries, in order to determine the routes of metastasis to the ovaries. Furthermore, we also investigated peritoneal dissemination during the first surgery, and lung or liver metastasis by MRI and CT. Furthermore, we also investigated the prognosis of patients with metastatic ovarian tumors and compared ovarian tumors treated simultaneously as the original cancers versus ovarian tumors treated after the original cancer.

 To study the vascular or lymphangio invasion of the ovary, we used several staining methods. We used the Victoria blue stain for investigating vascular invasion and immunohistochemical staining for D2-40 for lymphangio invasion. Positive portions for Victoria blue stain show elastic fibers of vessels, and positive portions for D2-40 show lymphatic endothelium. With respect to the Victoria blue stain, deparaffinized sections were immersed into 70% alcohol for 1 min, stained with Victoria blue solution (Muto Pure Chemical Co., Ltd, Tokyo, Japan) for 30 min, and they were then washed by water. After those procedures, the slides were stained by a routine hematoxylin and eosin method. With respect to the immunohistochemical examination for D2-40, we performed the avidin-biotin peroxidase complex method using a Vectastain Elite ABC kit (Vector, Burlingame, CA) according to the manufacturer's instructions. The sections were deparaffinized and boiled in a 10 mM citrate buffer in a microwave oven for 3 min. The sections were blocked for nonspecific binding and then incubated overnight with a mouse anti-D2-40 mAb (antipodoplanin monoclonal IgG, clone D2-40, Nichirei Bioscience INC, Tokyo, Japan) at 4°C [9]. The sections were then treated with a biotinylated horse antimouse immunoglobulin (Ig) G, followed by treatment with an avidin-biotin-peroxidase complex, and were finally stained with diaminobenzidine and 0.15% hydrogen peroxidase. Counterstaining was performed with Mayer's hematoxylin. 

Statistical analyses were performed using the chi-square test, Fisher's 2-tailed exact test, and Student's* t *test, on lymph invasion, vascular invasion, liver metastasis, pathological direct invasion, laterality, and prognosis.

## 3. Results

Eighteen cases of malignant tumors with metastasis to the ovary were identified. The average age of these patients was 58 years. The primary tumor sites were 7 gastric cancers, 6 colon cancers (2 ascending colons, 1 transverse colon, 1 sigmoid colon, and 2 rectums), 2 appendix, 1 small intestine, 1 gall duct, and 1 uterine endometrial cancer (Table 1).

**Figure 1:**
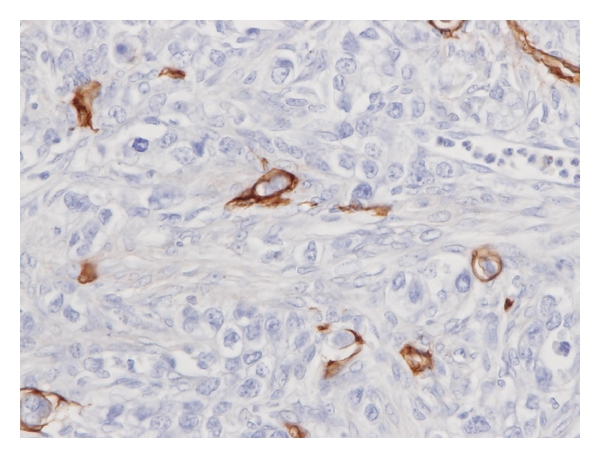
Immunohistochemical expression of D2-40 in metastatic ovarian tumor originating from gastric cancer. Immunohistochemical analysis revealed the tumor to be immunoreactive for D2-40. Positive portions (brown) show lymphatic endothelium. We found the cancer cells in the lymphatic vessels. It means positivity for lymphangio invasion.

We found 7 metastatic ovarian tumors originating from gastric cancers. The average age of these patients was 53 years. Five (71%) of the 7 gastric cancers had regional lymph node metastases (Table 2). Immunohistochemically, 4 cases (57%) had ovarian lymphangio invasion (Figure 1), and no one (0%) had ovarian vascular invasion. Two (29%) of the 7 gastric cancers had peritoneal dissemination. However, there were no cases of liver or lung metastases. With regard to the laterality of the metastatic ovarian tumor, 4 (57%) out of 7 were bilateral. Four (57%) of the 7 were treated at the same time as the original cancers, and 3 (43%) were treated later. All patients were dead within 1–7 years after treatment.

**Figure 2:**
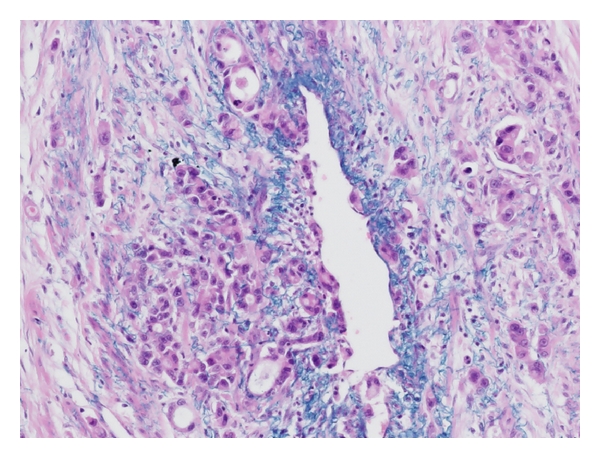
Victoria blue stain in metastatic ovarian tumor originating from ascending colon cancer. Positive portions (blue) show elastic fibers of vessels. We found the cancer cells in the vessels. It means positivity for vascular invasion.

We found 6 metastatic ovarian tumors originating from colorectal cancers. The average age of the patients was 64 years. Five (83%) of the 6 colorectal cancers had regional lymph node metastases (Table 3). Immunohistochemically, none had lymphangio invasion, but 4 (67%) had vascular invasion to the ovary (Figure 2). Furthermore, we found one patient with liver metastasis, which we did not experience in the gastric cancer cases. There were 2 (33%) direct invasion cases. Their origins were the sigmoid colon and rectum, which are near the ovaries.

With regards to other 5 metastatic ovarian tumors originating from nongenital (1 gall duct, 2 appendix, 1 small intestine) and genital organ (1 uterine corpus), the average age of these patients was 59 years. None of these 5 cases had ovarian lymphangio invasion or vascular invasion (Table 3). However, 4 (80%) of the 5 cases had peritoneal disseminations. In 2 (40%) of these 5 cases, we found direct pathological invasion to the ovaries. This was approximately the same value as invasion to the colorectal cancers (33%). Direct invasion was found in the ovarian tumors from the small intestine and uterine corpus, which were all near the ovaries. Since all of these organs were adjacent except for the gall duct, all cases except for the gall duct case were treated at the same time as the ovarian tumors.

 There were 4 (57%) ovarian lymphangio invasion cases in the 7 gastric cancers, but there was no such case (0%) in the 6 colorectal cancers (*P* < 0.05) (Table 3). There were no (0%) ovarian vascular invasion cases and no cases of liver metastasis in the 7 gastric cancers. On the other hand, there were 4 (67%) ovarian vascular invasion cases and one (17%) liver metastasis case in the 6 colorectal cancers. There were significant differences between them (*P* < 0.05). We hypothesized that there was a relatively higher rate of vascular metastasis in the colorectal cancers than in the gastric cancers.

 Direct pathological invasion into the ovary was observed at high frequency in the primary cancers with location near the ovaries (near the ovaries versus distant from the varies; 57% versus 0%, *P* < 0.05) (Table 3). However, in terms of the laterality of metastasis to the ovary, there was no significant difference between the primary cancers with location near the ovaries and distant primary cancers from the ovaries (57% versus 45%).

 Seventeen (94%) of the 18 patients were already dead despite intensive treatment. Since all of the patients were treated at advanced stage, they had poor prognoses. We studied the prognoses of those patients in whom we treated the metastatic ovarian tumors at the same time as the treatment for the original cancers versus later and in whom the primary cancers were near the ovaries or distant from the ovaries (Table 3). Patients with metastatic ovarian tumors originating from distant organs who were treated at the same time as the original cancers had a significantly poorer prognosis than patients with ovarian tumors treated later (time from primary treatment to death: 1.60 years versus 3.17 years, *P* < 0.05).

## 4. Discussion

Tumors can spread to the ovary by several pathways, such as direct spread, transcoelomic dissemination, hematogeneous spread, and lymphatic spread [10]. However, there are many cases with mixed metastatic pathways, because the original cancers are detected at advanced stage. It is very difficult to determine the specific pathway of tumor spread. However, it is possible to propose trends in the pathways of each primary cancer to the ovary using detailed clinicopathological examinations. 

In our study, the rate of lymphatic metastasis from the stomach to the ovary was significantly higher than from the colon to the ovary. The reason may be due to lymphatic vessel anatomy. Urogenital lymph vessel tracts give rise via the lumbar trunks to the receptaculum chili, which join the intestinal trunks. The intestinal trunks connect via celiac nodes to the gastric nodes, hepatic nodes, pancreaticolineal nodes, and mesenteric nodes (superior mesenteric and mesocolic nodes). Since the distance from the receptaculum chili to the gastric nodes is shorter than to the mesenteric nodes, gastric cancer cells metastasize easily via the receptaculum chili to the urogenital lymph vessel trunks, which supply the ovaries. Al-Agha and Nicastri also suggested that lymphatic spread was the most likely route of metastasis of gastric cancer to the ovaries [8]. Their opinion supports our hypothesis. 

In our study, the rate of vascular metastasis from the colo-rectum to the ovary was significantly higher than from the stomach to the ovary (using immunohistochemical methods). In addition, one had liver metastasis in the colorectal cancers. We suggest that the reason why is because the number, area, and volume of vessels in the colon are all larger than those of the stomach. Moore et al. reported that the route of metastasis from the colon to the ovary was suspected to be through hematogeneous pathways, because the laterality of the metastasis to the ovary in colon cancers did not correspond to the side of the primary lesion [11]. 

 Those cancers arising from origin of primary tumors with location near the ovaries were more likely to invade the ovaries directly than cancers arising from distant organs (*P* < 0.05). All of these organs and ovaries are intrapelvic tissues and thus are linked together by the peritoneum. However, the primary cancers with location near the ovaries do not always invade the ovary on the same side. There was no significant difference in metastatic laterality to the ovary between primary cancers with location near the ovaries and distant primary cancers from the ovaries in this study. When the primary cancers with location near the ovaries are present in the center of the pelvis or in appendix, then the cancers will likely invade to both ovaries. 

The absence of residual disease after treatment and a limited disease extent are favorable prognostic factors for metastatic ovarian tumors [12–15]. The amount of residual tumor after primary surgery is likely a prognostic factor [16, 17]. The prognoses of patients with metastatic ovarian tumors are generally poor, because the primary cancers are found at advanced stage [18, 19]. We therefore studied the differences in prognosis between time and space. Those patients with metastatic ovarian tumors originating from distant organs who were treated at the same time as the primary cancers had a significantly poorer prognosis than patients with ovarian tumors treated later (time from primary treatment to death: 1.60 years versus 3.17 years, *P* < 0.05). This means that the former patients had distant metastasis at the time of the first surgical treatment, whereas the latter patients did not have distant metastases at the time of their primary surgery. In brief, the patients with distant metastases at the time of primary surgery had a poorer prognosis than the patients without distant metastases.

## 5. Conclusion

The rate of lymphatic metastasis from the stomach to the ovary was significantly higher than from the colon to the ovary. In addition, we hypothesized that the rate of intravascular metastasis from the colo-rectum to the ovary was relatively higher than from the stomach to the ovary.

##  Conflict of Interests

The authors reported no potential conflict of interests.

## Figures and Tables

**Table 1:** The primary tumor sites of metastatic ovarian cancers.

Original organs	No.	%
Stomch	7	38.9
Colon	6	33.3
Ascending colon	2	11.1
Transverse colon	1	5.6
Sigmoid colon	1	5.6
Rectum	2	11.1
Appendix	2	11.1
Small intestine	1	5.6
Gall duct	1	5.6
Uterine corpus	1	5.6

Total	18	100

**Table 2:** Clinicopathologic findings and prognoses of the patients with metastatic ovarian cancers originating from gastric and colorectal cancers.

Patient no.	Age	Origin	Histology	Regional lymph node metastasis	Ovarian lymphangio invasion	Ovarian vascular invasion	Peritoneal dissemination	Direct invasion	Lung or liver metastasis	Laterality	Time of treatment for ovarian metastasis	Prognosis
1	26	Stomach	Poorly differentiated	O	X	X	X	—	X	Bilateral	At the same time	After 1.5 years (dead)
2	35	Stomach	Signet	X	O	X	X	—	X	Bilateral	One year later	After 2.0 years (dead)
3	50	Stomach	Poorly differentiated	O	X	X	X	—	X	Right	At the same time	After 1.0 years(dead)
4	51	Stomach	Signet	O	O	X	O	—	X	Bilateral	At the same time	After 2.4 years (dead)
5	64	Stomach	Moderately differentiated	O	O	X	X	—	X	Left	2 years later	After 3.0 years (dead)
6	70	Stomach	Poorly differentiated	O	O	X	O	—	X	Bilateral	At the same time	After 1.5 years (dead)
7	72	Stomach	Moderately differentiated	X	X	X	X	—	X	Left	4 years later	After 7.0 years (dead)

8	51	Ascending colon	Well differentiated	O	X	X	O	X	X	Right	3 years later	After 4.0 years (dead)
9	62	Ascending colon	Moderately differentiated	O	X	O	X	X	Liver	Right	One year later	After 1.5 years (dead)
10	70	Transverse colon	Moderately differentiated	O	X	O	O	X	X	Right	1.5 years later	After 1.5 years(dead)
11	67	Sigmoid colon	Moderately differentiated	X	X	O	X	O	X	Left	One year later	After 4.0 years (dead)
12	48	Rectum	Moderately differentiated	O	X	O	X	X	X	Bilateral	At the same time	After 2.0 years (dead)
13	84	Rectum	Moderately differentiated	O	X	X	X	O	X	Left	At the same time	After one year (alive)

(patient no. 14–18 (gall duct, appendix, appendix, small intestine, uterine corpus) are omitted).

**Table 3:** The pathways of metastases to the ovary and the differences in prognosis between time and space.

Site of original cancer	Ovarian lymphangio invasion	*P* value	Ovarian vascular invasion	*P* value	Liver metastasis	*P* value
Gastric cancer	57% (4/7)	*P* < 0.05	0% (0/7)	*P* < 0.05	0% (0/7)	NS
Colorectal cancer	0% (0/6)		67% (4/6)		17% (1/6)	

Site of original cancer	Pathological direct invasion	*P* value	Bilateral ovarian metastasis	*P* value		

Near the ovaries	57% (4/7)	*P* < 0.05	57% (4/7)	NS		
Distant from the ovaries	0% (0/11)		45% (5/11)			

Site of original cancer	Time of treatment for metastatic ovarian tumor	Number (without alive one)	Ovary-specific survival (years) (without alive one)	*P* value		

Near the ovaries	At the same time	3	2.67 ± 2.08			
Distant from the ovaries		7	1.60 ± 0.84*	*P < 0.05		
Near the ovaries	Later	1	4			
Distant from the ovaries		6	3.17 ± 2.11*			

Near the ovaries: sigmoid colon, rectum, appendix, small intestine, and uterine corpus, distant from the ovaries: stomach, ascending colon, transverse colon, and gall duct, ovary-specific survival: date of ovarian metastasis diagnosis to death.

## References

[1] Novak C, Gray LA (1938). Krukenberg tumor of the ovary: clinical and pathological study of four cases. *Surgery Gynecology and Obstetrics*.

[2] Young RH, Hart WR (1989). Metastases from carcinomas of the pancreas simulating primary mucinous tumors of the ovary. A report of seven cases. *American Journal of Surgical Pathology*.

[3] Young RH, Scully RE (1991). Metastatic tumors in the ovary: a problem-oriented approach and review of the recent literature. *Seminars in Diagnostic Pathology*.

[4] Khunamornpong S, Lerwill MF, Siriaunkgul S (2008). Carcinoma of extrahepatic bile ducts and gallbladder metastatic to the ovary: a report of 16 cases. *International Journal of Gynecological Pathology*.

[5] Kondi-Pafiti A, Kairi-Vasilatou E, Iavazzo C Metastatic neoplasms of the ovaries: a clinicopathological study of 97 cases.

[6] Okamoto T, Matsumura N, Mandai M (2011). Distinguishing primary from secondary mucinous ovarian tumors: an algorithm using the novel marker DPEP1. *Modern Pathology*.

[7] Parker RT, Currier JL, Coppleson M (1992). Metastatic tumors of ovary. *Gynecologic Oncology*.

[8] Al-Agha OM, Nicastri AD (2006). An in-depth look at Krukenberg tumor: an overview. *Archives of Pathology & Laboratory Medicine*.

[9] Kahn HJ, Bailey D, Marks A (2002). Monoclonal antibody D2-40, a new marker of lymphatic endothelium, reacts with Kaposi’s sarcoma and a subset of angiosarcomas. *Modern Pathology*.

[10] Young RH, Scully RE, Kurman RJ (1987). Metastatic tumors of the ovary. *Blaustein’s Pathology of the Female Genital Tract*.

[11] Moore RG, Chung M, Granai CO, Gajewski W, Steinhoff MM (2004). Incidence of metastasis to the ovaries from nongenital tract primary tumors. *Gynecologic Oncology*.

[12] Kim HK, Heo DS, Bang YJ, Kim NK (2001). Prognostic factors of Krukenberg’s tumor. *Gynecologic Oncology*.

[13] Cheong JH, Hyung WJ, Chen J, Kim J, Choi SH, Noh SH (2004). Survival benefit of metastasectomy for Krukenberg tumors from gastric cancer. *Gynecologic Oncology*.

[14] Lee SJ, Bae JH, Lee AW, Tong SY, Park YG, Park JS (2009). Clinical characteristics of metastatic tumors to the ovaries. *Journal of Korean Medical Science*.

[15] Sharma K, Rathi AK, Sood G (2008). Metastatic ovarian tumour in patient of carcinoma stomach. *Tropical Gastroenterology*.

[16] Petru E, Pickel H, Heydarfadai M (1992). Nongenital cancers metastatic to the ovary. *Gynecologic Oncology*.

[17] Kim WY, Kim TJ, Kim SE (2010). The role of cytoreductive surgery for non-genital tract metastatic tumors to the ovaries. *European Journal of Obstetrics Gynecology & Reproductive Biology*.

[18] Akhan SE, Kilic G, Salihoglu Y, Bengisu E, Berkman S (2001). Nongenital metastatic cancers of the ovary: a clinical analysis. *European Journal of Gynaecological Oncology*.

[19] Skírnisdóttir I, Garmo H, Holmberg L (2007). Non-genital tract metastases to the ovaries presented as ovarian tumors in Sweden 1990–2003: occurrence, origin and survival compared to ovarian cancer. *Gynecologic Oncology*.

